# Evaluation of the UC-TIB-LGV assay for the genotyping of *Chlamydia trachomatis* in anorectal samples

**DOI:** 10.1128/jcm.00060-26

**Published:** 2026-04-09

**Authors:** Arabella Touati, Béatrice Berçot, Cécile Bébéar, Olivia Peuchant

**Affiliations:** 1 Department of Bacteriology, CHU Bordeaux, National Reference Centre for Bacterial Sexually Transmitted Infections 36836, Bordeaux, France; 2 Department of Bacteriology Saint Louis-Lariboisière, APHP, Associate-Laboratory of the National Reference Centre for Bacterial Sexually Transmitted Infections, Paris, France; 3 Université de Bordeaux, CNRS Microbiologie Fondamentale et Pathogénicité (MFP), ARMYNE, Bordeaux, France; Marquette University, Milwaukee, Wisconsin, USA

**Keywords:** *C. trachomatis*, LGV, anorectal, diagnosis, assay

## LETTER

Lymphogranuloma venereum (LGV) is a sexually transmitted infection caused by *Chlamydia trachomatis* genovars L1 to L3. The identification of L genovars of *C. trachomatis* is of therapeutic interest because LGV treatment requires 3 weeks of doxycycline compared with 1 week for an infection with a non-L genovar (1).

According to the European guideline on the management of lymphogranuloma venereum (LGV), all men who have sex with men with a *C. trachomatis*-positive anorectal sample, irrespective of symptoms, should be tested for LGV (2). Only a limited number of commercially available single-plex or multiplex assays for LGV detection are currently available (3, 4).

The UC-TIB-LGV assay (Roche Diagnostics, Switzerland) has recently been developed for use on the open channel functionality (Cobas omni utility channel, CO) of the high-throughput Cobas x800 system (Roche Diagnostics). This assay is a fully automated *in vitro* nucleic acid amplification test, intended for use as a second-line diagnostic method for the specific detection of LGV infections from *C. trachomatis*-positive genital swabs or urine specimens collected in Cobas PCR media. It is based on a TaqMan real-time PCR targeting a region of the *pmp*H gene, commonly used by commercially available assays for LGV detection (3, 4), in which L genovars present a unique 36 bp gap (5). The UC-TIB-LGV assay can differentiate LGV and non-LGV strains or detect mixed infections as it contains one probe specific for L genovars and another for non-L genovars.

Because LGV is mainly responsible for anorectal infections, we evaluated the performance of the UC-TIB-LGV assay on 150 anorectal *C. trachomatis*-positive specimens collected in cobas PCR media between 2023 and 2025 at the French National Reference Centre (NRC) for bacterial sexually transmitted infections. This panel included 58 LGV and 92 non-LGV specimens. At the NRC, specific LGV detection was performed using an in-house real-time PCR assay targeting the *pmp*H gene (5).

Anorectal specimens collected in Cobas PCR media were directly loaded onto the Cobas 5800 system containing the CO reagent cassette filled with PCR mix and UC-TIB-LGV primers/probes. In case of discrepant results, sequencing of the *omp*A gene was performed (6).

The UC-TIB-LGV assay detected all 58 LGV-positive specimens and 87 out of 92 non-LGV specimens, showing an overall agreement of 96.6% (95% CI, 92.4–98.5) with our method and overall positive and negative agreement of 100% (95% CI, 93.8–100) and 98.8% (95% CI, 93.8–99.8), respectively (Table 1). The Kappa coefficient was 0.9. Among five non-LGV specimens, one gave a discrepant result and was detected as LGV by the UC-TIB-LGV assay but was negative by our real-time PCR and was confirmed as non-LGV by *omp*A gene sequencing. The remaining four were not detected by the UC-TIB-LGV assay but confirmed positive for *C. trachomatis* by the cobas CT/NG assay. Unfortunately, we failed to amplify the *omp*A gene for these four specimens, probably due to a low bacterial load. In six LGV-positive specimens, the UC-TIB-LGV assay was able to detect a mixture of LGV and non-LGV DNA.

**TABLE 1:** Clinical performance of the UC-TIB-LGV assay for the detection of LGV^*a*^

		NRC used method: In-house TaqMan real-time PCR	
		LGV (*n* = 58)	Non-LGV (*n* = 92)
Commercial kit UC-TIB-LGV	LGV	58	1
	Non-LGV	0	87
	NA	0	4

^*a*^ NA, no amplification; NRC, National Reference Center.

The UC-TIB-LGV assay showed good performance for the detection of LGV and non-LGV in *C. trachomatis*-positive anorectal specimens. One limitation of this study is that specimens collected in 2023 and 2024 had undergone one freeze/thaw cycle, which may have caused DNA degradation. However, all of these specimens still produced an interpretable result with the UC-TIB-LGV assay. The assay is research-use-only marked, but both the cassette and the cobas 5800/6800/8800 are IVDR-marked. The main limitation is that the manufacturer recommends specimen collection in cobas media, meaning that this assay is primarily useful for laboratories equipped with Roche detection systems.

In conclusion, the UC-TIB-LGV assay is a reliable method allowing, for the first time, the simultaneous detection of LGV and non-LGV directly from *C. trachomatis*-positive anorectal samples analyzed on automated cobas systems. Given the limited number of commercially available LGV detection assays, this new assay, designed for use on automated high-throughput PCR systems, will allow gain in diagnostic time and appropriate patient treatment.
